# The Responsibility of the Anæsthetist

**Published:** 1917-07-28

**Authors:** 


### THE RESPONSIBILITY OF THE ANÆSTHETIST.

In relation to a fatal issue occurring in the course of an operation there have at times been discussions as to the relative responsibilities of the surgeon on the one hand and the anæsthetist on the other. At one time certainly the latter was looked upon as the subordinate of the surgeon, and as obliged to act more or less under the operator's direction. But gradually the position of the anæsthetist has become more decidedly an independent one. The range of the agents administered has much increased, and the methods employed have been marked by greater complexity and refinement. Hence the judgment of the anæsthetist has had a wider field for enterprise, and his skill has received a larger recognition. To-day, therefore, in his own department he is supreme and his word has the force of law. With this position of authority there has been, necessarily, an increase of responsibility. Here as elsewhere the privilege of independence is accompanied by the burden of obligation. Some recent records seem to make it desirable to emphasise this position. We refer to incidents in which the anæsthetic, instead of being administered to the patient gradually and in the form of vapour, was driven in a liquid form into the patient's throat, with the inevitable risk of prompt and fatal poisoning. The immediate cause of such accidents was an incorrect adjustment of the tubes of the apparatus to the bottle containing the anæsthetic, and in one of the records it is stated that this adjustment was effected by a theatre attendant. No doubt the occurrence was the result of a blunder, but it was a blunder of an order too serious for classification as mere misadventure. If the negligence was not technically "criminal negligence," it certainly very nearly approached this condemnation. Whatever term be applied to the act of the "attendant," it is essential to keep in the forefront the contention, that for this act the anæsthetist must be regarded as responsible. He undertakes a certain duty, and if he chooses to delegate to a subordinate an essential part of this duty, it must be plainly written that he does so at his peril. No refinement or subtilty of argument can modify such a contention. The attendant may merit this, that, or the other punishment, but no demand of this order touches the position of the anæsthetist. He professes a full personal responsibility, and this he must meet, or, failing to do so, must accept for himself the penalty appropriate to carelessness, exhibited in a moment of supreme and urgent duty. We are in full sympathy with the tide of opinion which has given to the anæsthetist an improved and independent professional status. Necessarily there goes with this a measure of duty which cannot be discharged by a substitute or subordinate.

